# Necrotic Pulp With Crown Discoloration Associated With Orthodontic Treatment: A Case Report

**DOI:** 10.7759/cureus.42420

**Published:** 2023-07-25

**Authors:** Abdulrahman K Alshammari

**Affiliations:** 1 Department of Preventive Dentistry, College of Dentistry, University of Ha'il, Ha'il, SAU

**Keywords:** internal bleaching, endodontic treatment, internal resorption, necrotic pulp, orthodontic treatment

## Abstract

Orthodontic treatment may have iatrogenic consequences for the pulpal tissue. This study describes the endodontic treatment and internal bleaching that were used to treat a necrotic pulp with internal resorption caused by the dentist. This happened to the pulpal tissue after it had been treated with orthodontics. To prevent such iatrogenic consequences for pulpal tissue during orthodontic treatment, it is essential to maintain frequent radiological follow-ups. Regular radiographic examinations can help identify any potential complications early on, allowing for timely intervention and treatment. Additionally, employing light orthodontic force can help minimize the risk of trauma to the pulpal tissue, reducing the likelihood of necrosis and internal resorption.

## Introduction

The dental and periodontal tissues (PDL) undergo remodeling alterations when orthodontic force is used to move teeth [1]. The PDL and alveolar bone are compressed on one side and stretched on the other by the orthodontic force that is being used. As bones and the surrounding periodontal tissues are subjected to varying levels of orthodontic force in terms of intensity, frequency, and duration, there are noticeable microscopic modifications that occur [1-3]. The process of receiving orthodontic treatment may have negative implications. These negative impacts include pulpal changes, root resorption, and periodontal disease [4].

Orthodontic tooth movement can cause biological, molecular, and physiological changes to the dental pulp. Among the effects are neurovascular abnormalities, the development of an inflammatory response, degenerative changes, altered pulpal sensibility, and reduced pulpal blood flow sensation [5,6]. Molecular changes include the delivery of angiogenic and vascular endothelial growth factors, a decrease in alkaline phosphatase activity, growth in aspartate aminotransferase activity, and an increase in the amount and width of microvessels [5]. The physiology of the tooth pulp may change due to a variety of circumstances, such as patient age, apical root maturation, and orthodontic mechanics [7]. Physiological alterations in the pulp raise the sensitivity thresholds for electrical stimulation. According to a previous study, using orthodontic force for a month decreased reaction thresholds and decreased the reaction to the pulp tester [8].

Immature teeth are less prone to sustain damage because of the richer, larger, and thicker neurovascular bundle supplying the tooth [9]. A light orthodontic force must be used in order to effectively move teeth, prevent dental pulp damage, and alleviate any damage that results from orthodontic treatment [9]. Although pulpal necrosis or dental pulp obliteration are not caused directly by orthodontic tooth movement, a prior dental trauma may be the underlying cause if these problems are noticed after orthodontic tooth movement [10,11]. The prevalence of pulpal necrosis in teeth that had previous dental trauma was reported to be 9.1% and 10.4%, respectively, and occurs between 0.3% and 0.5% of the time in teeth without dental trauma [12,13].

External root resorption is a frequent occurrence during orthodontic tooth movement and may be considered a side effect of orthodontic therapy [1,14]. The etiology of root resorption is currently unknown and complicated, involving genetic predisposition as well as environmental variables [15,16]. The duration of orthodontic treatment, as well as thin, tapered, and dilacerated roots, are all risk factors for root resorption [17-19]. Furthermore, a history of anterior tooth trauma increases the probability of root resorption [20]. Internal root resorption (IRR) is a pathological condition in which the dentin and odontoblast layer are eroded by the action of multinuclear cells with odontoclastic action toward the outer surface of the crown or root [21-23]. The etiology and pathology of IRR remain unknown. However, causing factors have been proposed to include trauma, surgical operations, intense pressure on an impacted tooth, mechanical, chemical, or thermal traumas, as well as persistent infection and inflammation in the pulp or periodontal tissues. This study outlines the root canal therapy and internal bleaching that were used to treat a necrotic pulp linked with internal resorption that occurred after fixed orthodontic appliance treatment.

## Case presentation

**Figure 1:**
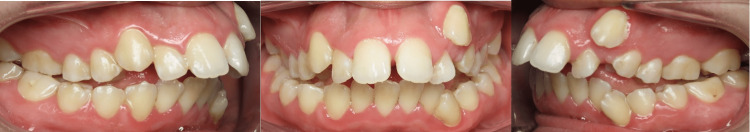
Pretreatment intraoral photographs.

A 23-year-old girl came to an orthodontic clinic in the private sector. The chief concern was the crowding in the maxillary teeth. She had no history of trauma to the frontal mouth region, and she was in good physical health, so the medical history was not relevant. The patient had a severe skeletal class II malocclusion with a retrognathic mandible (Table 1). An intraoral examination revealed an Angle class II malocclusion with proclined maxillary and mandibular incisors, moderate crowding in the maxillary and mandibular arches, and a narrow maxillary arch with bilateral posterior crossbite (Figure 1).

**Table 1:** Pretreatment cephalometric analysis. SNA: sella-nasion-A-point (angle); SNB: sella-nasion-B-point (angle); ANB: A-point-nasion-B-point (angle); FMA: Frankfort-mandibular (plane) angle; ODI: overbite depth indicator; IMPA: incisor-mandibular plane angle; NLA: nasolabial angle; NA: nasion to point A; NB: nasion to point B

Parameter	Pre-treatment	
SNA	Skeletal	75.31°
SNB		62.86°
ANB		12.46°
Wits appraisal		10.93°
FMA		47.78°
Gonial angle		130.18°
ODI		72.47°
U1 to NA linear	Dental	8.59 mm
U1 to NA angular		27.64°
L1 to NB linear		17.17 mm
L1 to NB angular		46.12°
IMPA		101.85°
NLA	Soft tissue	90.95°
Upper lip to E-plane		8.35 mm
Lower lip to E-plane		11.63 mm

**Figure 2:**
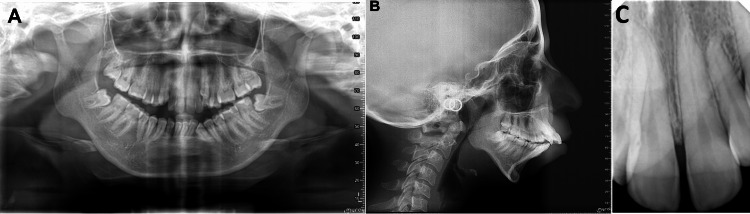
Pretreatment radiographs - (A) panoramic, (B) cephalometric, and (C) periapical.

The panoramic radiograph showed an asymmetric condyle with mesioangular impacted teeth #38 and #48 (Figure 2A). A cephalometric radiograph showed severe skeletal class II malocclusion with retruded both maxilla and mandible with a hyperdivergent skeletal pattern and proclined and protruded both maxillary and mandibular incisors (Figure 2B). A periapical x-ray showed normal tooth structure without any periapical lesion or loss of periodontal ligament for maxillary incisors (Figure 2C).

The treatment plan was considered as follows: (i) orthodontic treatment with all first premolars extracted only to align the maxillary and mandibular teeth and accept the skeletal discrepancy, (ii) orthodontic treatment with all first premolar extraction and maxillomandibular orthognathic surgery with genioplasty to correct the skeletal and dental malocclusion, (iii) orthodontic treatment with all first premolars extracted followed by genioplasty to improve the facial profile after discussing the available alternatives with the patient, it was decided to combine orthodontic treatment with orthognathic surgery.

**Figure 3:**
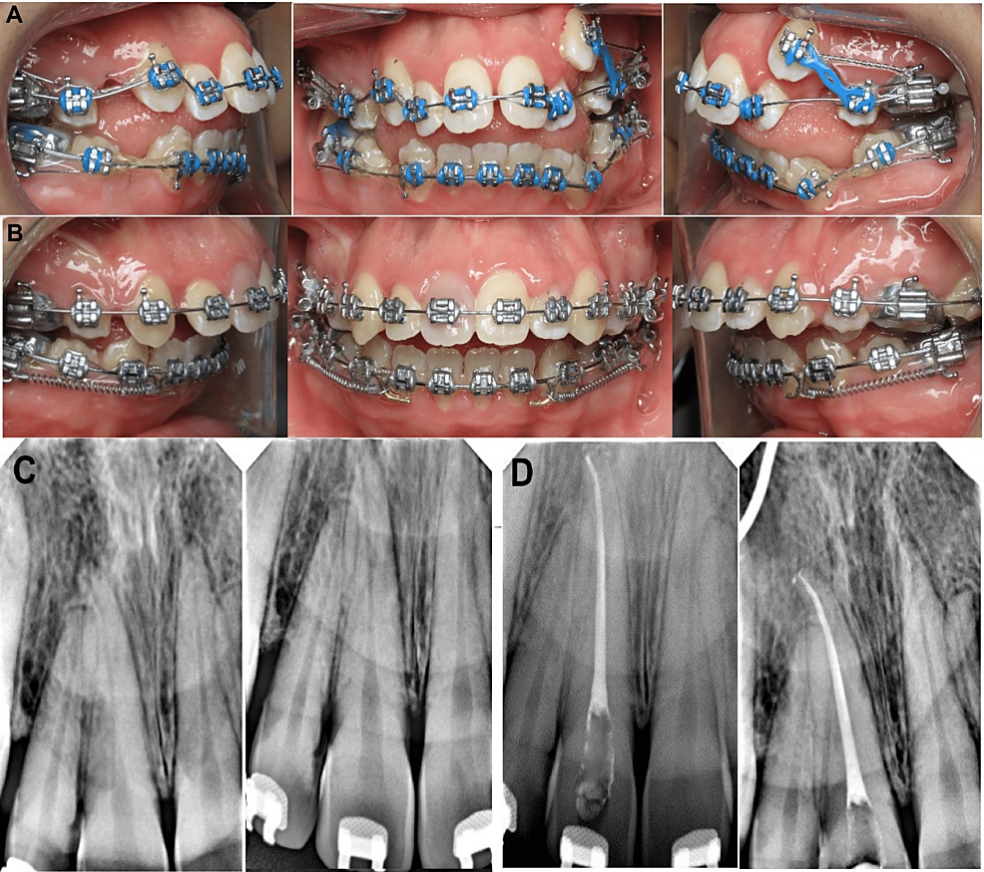
Intraoral photograph during orthodontic treatment. (A) After four months of treatment, (B) crown discoloration of maxillary right central incisors after eight months of treatment, (C) preoperative radiograph of the maxillary right central incisor revealing a periapical radiolucency with widening periodontal ligaments, and (D) postoperative radiograph after root canal treatment of maxillary right central incisor.

Treatment was started by bonding the fixed orthodontic appliances for both maxillary and mandibular teeth using 0.022×0.028 inch slot straight wire appliances with McLaughlin-Bennett-Trevisi (MBT) prescription (Gemini; Monrovia, CA: 3M Unitek). For anchorage, the transpalatal arch (TPA) was used with molar bands. A 0.014-inch nickel-titanium (NiTi) archwire was engaged through the brackets except for the maxillary left canine (23) because it was displaced high labially. After two months, a 0.016-inch (NiTi) archwire was inserted to continue the alignment of the teeth. An intraoral photograph taken after four months of bonding the braces showed tooth number 11 with normal color (Figure 3A). After that, a 0.017×0.025 inch (NiTi) archwire was inserted in the bracket slot to continue the alignment. When the teeth were aligned, a 0.016×0.022 inch stainless steel (SS) archwire was applied as a base archwire to preserve the maxillary arch form, whereas a 0.012-inch nickel-titanium (NiTi) archwire was inserted through the brackets of the displaced canine (23) by the piggyback technique. When tooth (23) reached the occlusion, a 0.016-inch (NiTi) archwire was engaged to align tooth (23) in the arch. Following receiving therapy for 10 months, an intraoral examination indicated that the maxillary right central incisor's labial and palatal surfaces of the crown were discolored (Figure 3B). The tooth had no caries, periodontal pockets, or restorations. The tooth displayed a slight degree of mobility. Electric pulp tests (Vitality Scanner; Glendora, CA: Analytic Technology) and cold testing revealed no abnormalities in any of the maxillary incisors (Endo-ice; Akron, OH: The Hygienic Corporation), except tooth #11. Radiographic examination revealed a radiolucent apical area for tooth #11 with widening periodontal ligaments of the tooth (Figure 3C). The root canal procedure for tooth 11 was scheduled after a necrotic pulp diagnosis. In order to apply clamps and rubber dams more easily, braces were taken off of the four maxillary incisors. The coronal access was accomplished using burs that covered the complete resorptive coronal defect; a working length was determined, and the intracanal tissue was removed while being heavily irrigated with a solution of 2.5% sodium hypochlorite. Debridement of the root canal was then performed, with a working length of 25.5 mm. The root canal was cleaned and shaped, then dried and completely filled with gutta-percha. The pulp chamber was restored using the CavitTM (Saint Paul, MN: 3M ESPE) during the same visit (Figure 3D).

**Figure 4:**
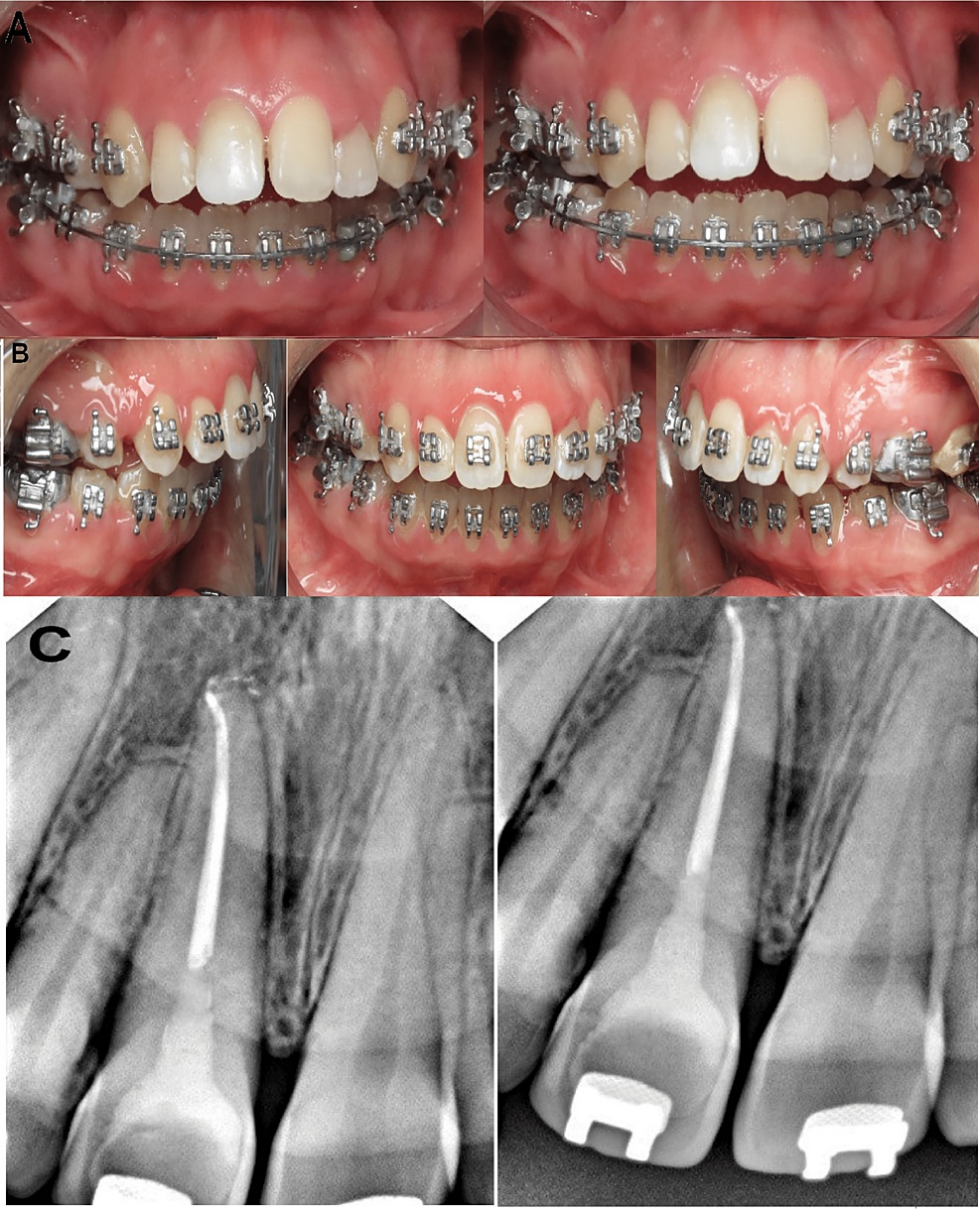
Follow-up photographs and radiographs after treatment. The images show (A) treated tooth after internal bleaching, (B) treated tooth after one year of internal bleaching without any relapse, and (C) radiograph of treated tooth after one year following up without any signs of internal resorption or any radiolucency.

Internal bleaching with Whiteness Super-endo (37% Opalescence Endo; South Jordan, UT: Ultradent) was applied to the tooth three months later to whiten the tooth discoloration. In order to rebuild the pulp chamber, photopolymerized composite resin was used (Figures 4A-4C).

**Figure 5:**
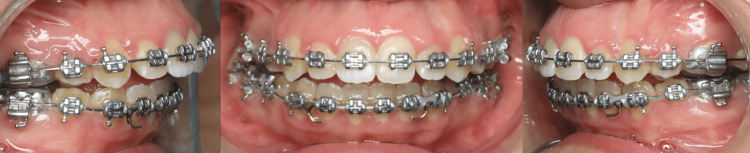
Two years follow-up after treatment of tooth #11.

Orthodontic treatment was resumed after bleaching. The subject underwent clinical and radiographic examinations at intervals of one month, three months, six months, and one year following treatment. The clinical and radiographic evaluations were carried out by a dentist who was also treating the patients at the time of the visit. After two years of follow-up, clinical and radiographical assessment of tooth #11 revealed that there had been no color regression and that the tooth was asymptomatic and free of periodontal issues (Figure 5).

## Discussion

Previous trauma could be the reason for necrotic pulp and internal resorption [24,25]. The application of excessive, uncontrolled force by the orthodontist is another likely factor [26]. Clinical examination in the current case revealed that tooth #11 was healthy and unrestored, making them important before treatment. Moreover, the patient denied having ever experienced dental trauma.

Orthodontic iatrogenic factors may have a major negative effect on oral health. These factors include impact on oral hygiene, risk of tooth decay, periodontal disease, inflammation, cellular and molecular alterations in the dental pulp, and root resorption. Root resorption occurs often during orthodontic tooth movement, and probable causes include both inherited and environmental factors [16,27-29]. After 10 months of therapy, in this case, the right central maxillary incisor showed minor internal resorption. Orthodontic forces have been demonstrated to result in cellular damage, inflammatory changes, and circulatory disruptions in the tooth pulp in addition to mechanical damage and inflammatory reactions in the periodontium [29,30]. Küçükkeleş and Okar reported that when teeth were exposed to intrusive force, the inner root surface of the pulp generated internal resorption [31]. Root resorption could also occur if the duration of orthodontic treatment exceeds 30 months [32]. However, the patient was in the leveling and alignment stage at that time, and no direct intrusive force was applied to the tooth. The necrotic pulp and root resorption most likely occurred after the treatment began. Unfortunately, no diagnostic radiograph was taken throughout the treatment. A follow-up radiography check could have detected the problem early [32]. These findings emphasize the necessity of regular patient monitoring during orthodontic treatment as a means of detecting and treating any complications at an early stage.

Discoloration of the anterior teeth may reduce a person's attractiveness. When compared to veneers or crowns, tooth whitening or bleaching is a good non-invasive therapy. In this clinical case, intrinsic factors induced discoloration of the tooth in the right maxillary central incisor. Intra-pulpal bleeding and erythrocyte lysis may have been caused by strong orthodontic force. A discolored tooth crown results from the subsequent bleeding that diffusely reaches the dentinal tubules. The length of necrosis in the pulp determines the degree of discoloration; the longer the colored components are present in the pulp, the more discoloration there is [33]. If internal resorption is left untreated, it may lead to root canal wall perforation, complicating the treatment [34]. Compared to porcelain veneers or crowns, teeth whitening is a less invasive, conservative, and aesthetically pleasing option. The oxidizing substance inside the pulp chamber comes into close contact with dentine during the non-vital teeth whitening process. Sodium perborate, carbamide peroxide, and hydrogen peroxide are the most often used internal bleaching agents [35-37]. Glass ionomer is utilized as the cervical seal or barrier to cover endodontic obturation. The barrier is positioned 1 mm under the cementoenamel junction and has a thickness of roughly 2 mm in order to stop internal bleaching and adverse effects, such as external root resorption, from happening [38-40]. The efficacy of intracoronal bleaching in teeth that have undergone endodontic therapy depends on the etiology, diagnosis, and choice of the best bleaching procedures [41]. It was decided to employ the walking bleach method since it is quicker, safer, and more comfortable for the patient. The active ingredient is placed in the pulp chamber, and the tooth canals are subsequently sealed as part of the walking bleach procedure. In this case, bleaching materials were applied and replenished for two sessions, and the coronal restoration was completed with a composite restoration.

The type of resorption, its cause, the inflammatory process, and the severity of the lesion all affect how to manage unvital pulp tissue or resorbed roots [34]. If tooth root resorption is detected early and the sizes are acceptable, the case can be treated conservatively with endodontic root canal treatment and whitening. If the condition is severe, the second alternative is to extract the tooth and plan a prosthodontic treatment [34,42]. In this case, we chose conservative treatment since the tooth had a good prognosis and the size of the lesion was small.

Class II malocclusion can be treated using a variety of methods, including orthognathic surgery, growth modification, and orthodontic camouflage [43]. The timing of treatment, the severity of skeletal and dental abnormalities, and patient compliance all play a role in the treatment decision. In this case, growth modification and orthodontic camouflage were not viable options due to the patient being an adult and the presence of severe skeletal problems. The patient presented with a severe class II malocclusion, retrognathic mandible, increased overjet, and moderate crowding of the upper and lower teeth with proclined incisors. Therefore, the treatment plan involved fixed orthodontic appliances combined with orthognathic surgery to address the skeletal problem and achieve a class I relationship for both skeletal and dental factors. It has been reported in the literature that orthognathic surgery is often necessary for successful correction of malocclusion when the overjet exceeds 10 mm [44]. Additionally, older patients with more severe class II problems typically require surgical treatment [45]. The extraction of all first premolars was performed to alleviate crowding and normalize incisor proclination. Camouflage treatment was not considered feasible due to the severity of the skeletal problem. An alternative option would have been to treat only the maxillary arch after extracting the first premolars to address crowding and align the teeth. However, with this alternative option, the skeletal problems and increased overjet would have remained unchanged.

## Conclusions

Orthodontic treatment may have iatrogenic consequences for the pulpal tissue. This case report explains the need for routine patient radiological follow-up throughout orthodontic therapy by showing how necrotic pulp with internal resorption can appear at variable intervals after orthodontic therapy and could result in the degeneration of mineralized tissues. Furthermore, conventional endodontic therapy and an internal bleaching agent can be used to treat necrotic pulp and internal resorption to produce positive cosmetic and functional results.
